# Comprehensive Analysis of bHLH Transcription Factors in *Ipomoea aquatica* and Its Response to Anthocyanin Biosynthesis

**DOI:** 10.3390/ijms24065652

**Published:** 2023-03-15

**Authors:** Zheng Liu, Xiaoai Fu, Hao Xu, Yuxin Zhang, Zhidi Shi, Guangzhen Zhou, Wenlong Bao

**Affiliations:** 1 Key Laboratory for Quality Regulation of Tropical Horticultural Crops of Hainan Province, School of Horticulture, Hainan University, Haikou 570228, China; 2 College of Tropical Crops, Hainan University, Haikou 570228, China; 3 Hainan Yazhou Bay Seed Laboratory, Sanya Nanfan Research Institute of Hainan University, Sanya 572025, China

**Keywords:** gene family, cis-acting element, gene duplication events, evolutionary relationship, gene expression

## Abstract

The basic helix-loop-helix (bHLH) proteins compose one of the largest transcription factor (TF) families in plants, which play a vital role in regulating plant biological processes including growth and development, stress response, and secondary metabolite biosynthesis. *Ipomoea aquatica* is one of the most important nutrient-rich vegetables. Compared to the common green-stemmed *I. aquatica*, purple-stemmed *I. aquatica* has extremely high contents of anthocyanins. However, the information on *bHLH* genes in *I. aquatica* and their role in regulating anthocyanin accumulation is still unclear. In this study, we confirmed a total of 157 *bHLH* genes in the *I. aquatica* genome, which were classified into 23 subgroups according to their phylogenetic relationship with the bHLH of *Arabidopsis thaliana* (AtbHLH). Of these, 129 *IabHLH* genes were unevenly distributed across 15 chromosomes, while 28 *IabHLH* genes were spread on the scaffolds. Subcellular localization prediction revealed that most IabHLH proteins were localized in the nucleus, while some were in the chloroplast, extracellular space, and endomembrane system. Sequence analysis revealed conserved motif distribution and similar patterns of gene structure within *IabHLH* genes of the same subfamily. Analysis of gene duplication events indicated that DSD and WGD played a vital role in the *IabHLH* gene family expansion. Transcriptome analysis showed that the expression levels of 13 *IabHLH* genes were significantly different between the two varieties. Of these, the *IabHLH027* had the highest expression fold change, and its expression level was dramatically higher in purple-stemmed *I. aquatica* than that in green-stemmed *I. aquatica*. All upregulated DEGs in purple-stemmed *I. aquatica* exhibited the same expression trends in both qRT-PCR and RNA-seq. Three downregulated genes including *IabHLH142*, *IabHLH057*, and *IabHLH043* determined by RNA-seq had opposite expression trends of those detected by qRT-PCR. Analysis of the cis-acting elements in the promoter region of 13 differentially expressed genes indicated that light-responsive elements were the most, followed by phytohormone-responsive elements and stress-responsive elements, while plant growth and development-responsive elements were the least. Taken together, this work provides valuable clues for further exploring *IabHLH* function and facilitating the breeding of anthocyanin-rich functional varieties of *I. aquatica*.

## 1. Introduction

The bHLH proteins comprise one of the largest transcription factor (TF) families in eukaryotes including plants, animals, and fungi. The bHLH TF contains two functional conserved domains with a total of approximately 60 amino acid residues, namely the N-terminal basic region composed of 13–17 amino acids and the C-terminal HLH region with 40–50 amino acids, respectively. Typically, the basic region of bHLH TF in plants possesses a highly conserved HER motif that can specifically bind to the cis-acting element E-box (CANNTG) or G-box (CACGTG) within the promoter region of the target gene. The HLH region consists of two relatively conserved amphipathic α-helices that are separated by an intervening loop of variable length, which is required for the formation of bHLH dimers through protein-protein interaction to modulate the expression of downstream genes that correlated with diverse signaling pathways [1,2,3,4]. With the increasing publications of plant genome data, bHLH TFs in numerous plant species have been identified at the whole genome level. For instance, there are 602 *bHLH* genes in the *Brassica napus* genome [5], 208 in the *Zea mays* genome [6], 162 in the *Arabidopsis thaliana* genome [7], 115 in the *Vitis davidii* genome [8], and 141 in the *Hordeum vulgare* genome [9]. The bHLHs exert a broad range of functions in various biological processes of diverse plant species [3,10,11]. For instance, *Arabidopsis* flowering bHLHs are involved in the regulation of flowering time by activating the gene encoding CONSTANS (CO) protein [12]. Rice *bHLH* gene *INCREASED LAMINA INCLINATION1* (*ILI1*) participates in brassinosteroid-mediated plant development by serving as a downstream target of BRASSINAZOLE-RESISTANT1 (BZR1) TF [13]. In apple, MdbHLH33 facilitates plant cold stress tolerance and anthocyanin accumulation by positively regulating the expressions of the C-repeat binding factor (CBF) TF gene and dihydroflavonol 4-reductase (DFR) gene [14].

Anthocyanins are ubiquitous secondary metabolites generated from the branch of the phenylpropanoid biosynthesis pathway. As water-soluble pigments, anthocyanins are widely distributed in diverse plant organs and tissues and give rise to colors varying with pH value, playing a crucial role in plant pollination and seed dispersal. As natural antioxidants, anthocyanins can help plants defend against various stresses including drought, low temperature, and ultraviolet radiation [15,16]. Consuming a diet high in anthocyanins can enhance immunity and delay the aging process in humans [17]. The key structural genes and regulatory genes involved in the hierarchical regulatory networks governing the anthocyanin biosynthetic pathway are well-studied in many plant species [18,19]. The structural genes involved in the anthocyanin biosynthetic pathway are classified into two categories, namely the early biosynthetic genes (EBGs) and late biosynthetic genes (LBGs). Enzymes encoded by EGBs are responsible for producing large amounts of anthocyanin precursors while those encoded by LBGs are involved in the generation of various anthocyanins [20,21]. Studies showed that the expression of the EBGs is mainly activated or repressed by the MYB TFs, while the LBGs are coordinately modulated by the members of the MYB, bHLH, and WDR TF families [22,23,24]. Of these TFs, the bHLHs act as an essential cofactor of the MYBs and WDRs and play a central role in the late stage of anthocyanin biosynthesis.

Since the first bHLH protein associated with anthocyanin biosynthesis was discovered in maize, a growing understanding of bHLH functions regulating anthocyanin production in plants was carried out. In *Arabidopsis thaliana*, a bHLH TF TRANSPARENT TESTA8 (TT8) regulates the expression of the LBG *DFR* and *BANYULS* (*BAN*) genes which in turn modulate the seed coat pigmentation [25]. In *Chrysanthemum morifolium*, CmbHLH2 serves as the essential partner of CmMYB6 to facilitate the expression of *CmDFR* and thereby enhance anthocyanin accumulation [26]. In *Malus domestica*, the *MdbHLH3* gene was induced by low-temperature stress and promoted fruit coloration by activating the anthocyanin biosynthetic genes (ABGs) including *MdUFGT* and *MdDFR* [27]. In contrast, the *StbHLH1* gene of *Solanum tuberosum* was down-regulated under high-temperature stress and led to the reduction of anthocyanin contents in flesh by affecting the expression of the ABGs [28]. In *Populus*, the *PdbHLH* (a homolog of TT8 in *Arabidopsis*) acts as an efficient enhancer of PdMYB118 to promote the expression of the ABGs and thereby promotes the anthocyanin production under wound [29]. In *Vitis vinifera*, the VvMYC1 that has been characterized as bHLH TF cooperates with several MYB TFs to participate in the transcriptional cascade involved in the regulation of anthocyanin accumulation in berries [30]. In mulberry fruits, the *bHLH3* was implicated in maintaining flavonoid homeostasis, which regulated the fruit coloration by affecting the anthocyanin compositions [31]. These studies demonstrated that bHLH TFs act as vital regulators of anthocyanin biosynthetic pathways in diverse plant species.

*I. aquatica* has a wide distribution in tropical and subtropical regions. As a health-promoting leafy vegetable, *I. aquatica* is rich in essential amino acids, flavonoids, and diverse mineral elements such as calcium, potassium, and phosphorus [32,33]. Moreover, *I. aquatica* was used in traditional medicine as a remedy for different diseases including liver disorders, diabetes, and in the treatment of heavy metal intoxication [34,35,36]. Our previous study revealed that the purple-stemmed *I. aquatica* has extremely high anthocyanin contents relative to the common green-stemmed *I. aquatica* [37]. To understand the possible roles of bHLH TF related to anthocyanin biosynthesis in *I. aquatica*, we comprehensively analyzed the members of the *bHLH* gene family and their expression patterns by combining bioinformatics and experimental methods. Our study laid a foundation for further exploring the roles of *IabHLHs* in regulating anthocyanin biosynthesis in *I. aquatica*.

## 2. Results

### 2.1. Identification of IabHLH Genes in the I. aquatica Genome

**Figure 1:**
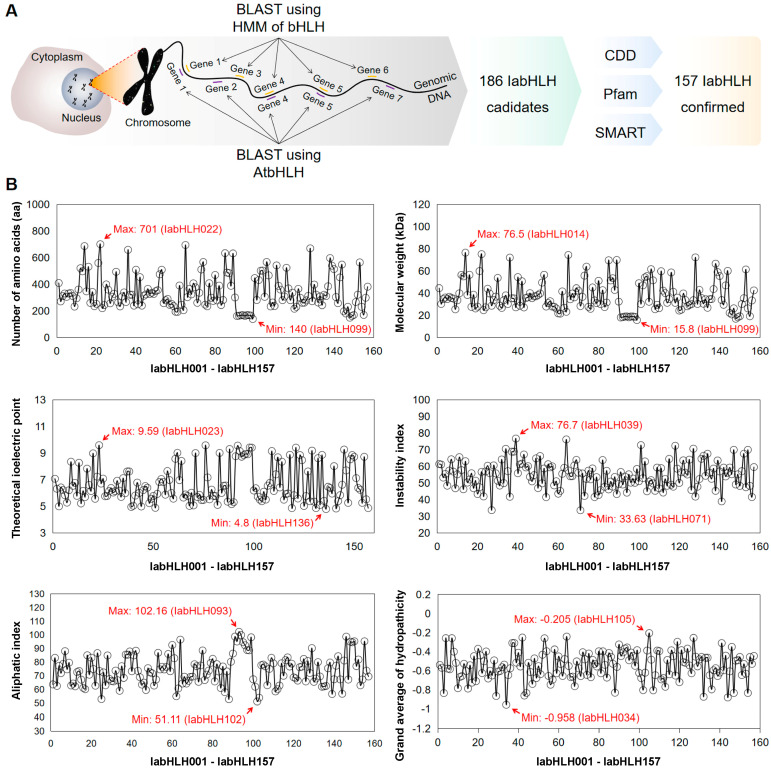
Identification of *IabHLH* genes. (**A**) Schematic diagram of the procedure for retrieving *IabHLH* genes from the *I. aquatica* genome. (**B**) The physicochemical properties of 157 IabHLH proteins.

To identify the members of the *IabHLH* gene family, the HMM profile of bHLH and the AtbHLH protein sequences were used as queries to search against the *I. aquatica* genome. After combining the above results, the redundant sequences and the sequences without the bHLH domain were eliminated based on the running consequences of Pfam, CDD, and SMART programs (Figure 1A). A total of 157 *IabHLH* genes were identified, which were named IabHLH001–IabHLH157 according to their positions on the chromosomes and scaffolds (Table S1). The physicochemical properties of proteins play a crucial role in the bio-function and conformation of proteins. As shown in Figure 1B and Table S2, the length of IabHLHs ranged from 140 aa (IabHLH099) to 701 aa (IabHLH022), and their molecular weight (MW) varied from 15.8 kDa (IabHLH099) to 76.5 kDa (IabHLH014). The IabHLH023 had the highest theoretical isoelectric point (pI), with a value of 9.59, while IabHLH136 had the lowest, with a value of 4.8. Except for IabHLH027, IabHLH071, and IabHLH141, the remaining IabHLHs had instability index (Ii) values greater than 40. In addition, 66 of the 157 IabHLHs had aliphatic index (Ai) values lower than 71. Analysis of the grand average of hydropathicity index (GRAVY) showed that all IabHLHs had negative GRAVY values. Subcellular localization prediction of IabHLHs showed that 139, 13, 4, and one bHLH were localized in the nucleus, chloroplast, extracellular space, and endomembrane system, respectively (Table S2).

### 2.2. Synteny Analysis of IabHLHs

**Figure 2:**
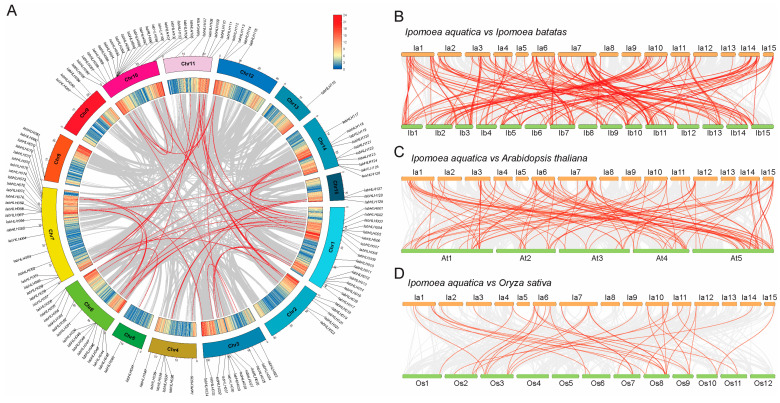
Analysis of *IabHLH* genes at the chromosome level. (**A**) Chromosomal location of the *IabHLH* genes. The gene pairs linked by red lines were the 44 *IabHLH* collinear gene pairs located on chromosomes; the outer circle represents 15 chromosomes of *I. aquatica*, while the inner circle indicates the gene density of each chromosome. (**B**–**D**) Syntenic relationships between *IabHLH* genes and the other plant species’ genome. The gray lines among chromosomes indicate all homologous gene pairs at the whole-genome level, while the red lines among chromosomes show homologous gene pairs related to the *IabHLH* genes. More information concerning the homologous gene pairs was shown in Table S3.

To understand the *IabHLH* gene distribution in the *I. aquatica* genome, their genomic location was analyzed. A total of 129 of the 157 *IabHLH* genes were unevenly distributed on 15 chromosomes, while the remaining genes were spread on the scaffolds (Figure 2A and Table S1). 

To reveal the *IabHLH* collinear gene pairs in the *I. aquatica* genome, the analysis of intraspecific collinearity was performed. A total of 61 *IabHLH* colinear gene pairs were identified in the *I. aquatica* genome, of which 44 gene pairs were mapped on the chromosomes (Figure 2A). To explore the potential evolutionary process of *IabHLH* genes, we conducted the analysis of interspecific syntenic relationships among *IabHLH* genes and the other plant species’ genomes, including plants of the *Convolvulaceae* family (*Ipomoea batatas*), *Brassicaceae* family (*A. thaliana*), and *Poaceae* family (*Oryza sativa*). As shown in Figure 2B, the highest number of homologous gene pairs were identified between *IabHLH* genes and *I. batatas*, with 239, indicating the closest evolutionary distances between the *IabHLH* gene family and *I. batatas*. As shown in Figure 2C,D, a total of 127 and 43 homologous gene pairs were identified between *I. aquatica* and *A. thaliana*, and between *I. aquatica* and *O. sativa*, respectively, suggesting a closer evolutionary relationship of the *IabHLH* gene family with *A. thaliana* than with *O. sativa*. Moreover, 26 *IabHLH* genes were shared among all homologous gene pairs (Table S3), inferring their relative conservation in the plant evolutionary process.

### 2.3. Phylogenetic Relationships of IabHLHs and AtbHLHs

**Figure 3:**
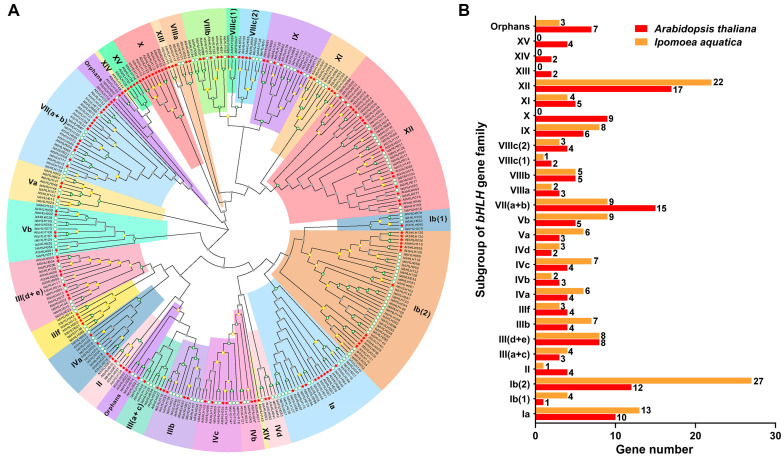
Phylogenetic relationships of IabHLHs and AtbHLHs (**A**) The phylogenetic tree contains 157 IabHLH proteins and 148 AtbHLH proteins. The hollow green circles represent IabHLHs, while the solid red pentagons represent AtbHLHs. The gray, yellow, and green squares represent the bootstrap values varied from 0 to 49, 50 to 79, and 80 to 100, respectively. (**B**) The number of IabHLHs and AtbHLHs in each subfamily.

To gain further insights into the potential biological functions of IabHLHs, the phylogenetic relationships of IabHLHs and well-characterized AtbHLHs were analyzed. The topology of the phylogenetic tree indicated that the *IabHLH* genes could be clustered into 23 subfamilies, including Ia, Ib(1), Ib(2), II, III(a+c), III(d+e), IIIb, IIIf, IVa, IVb, IVc, IVd, Va, Vb, VII(a+b), VIIIa, VIIIb, VIIIc(1), VIIIc(2), IX, XI, XII, and orphans. The IabHLHs were not clustered together with AtbHLHs in the subfamilies X, XIII, XIV, and XV. In addition, 3 IabHLH orphans were not gathered in the same node (Figure 3A). The subfamily Ib(2) was the largest, with 27 *IabHLH* genes, followed by subfamily XII, with 22 *IabHLH* genes, while the subfamily II and VIIIc(1) were the smallest, with only one *IabHLH* gene (Figure 3A).

### 2.4. The Sequence Features of the IabHLHs

**Figure 4:**
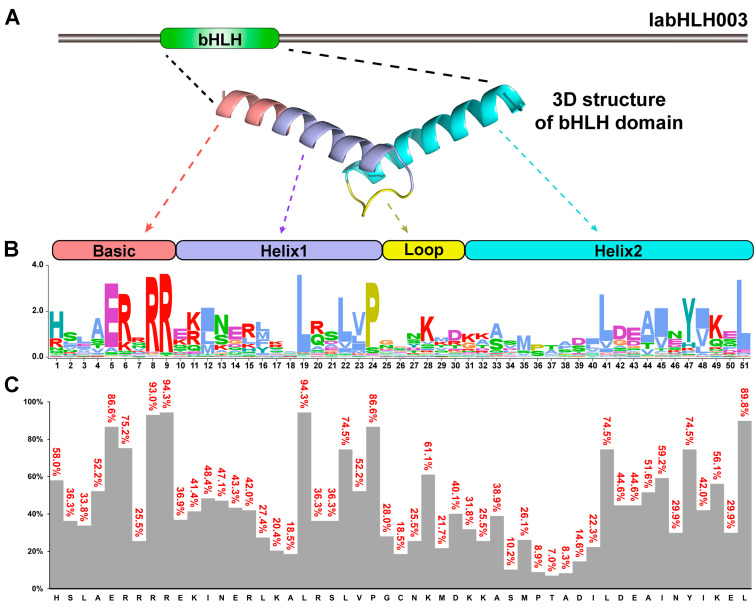
The sequence characteristics of IabHLHs. (**A**) Three-dimensional structure of bHLH domain in the IabHLH003. (**B**) Sequence logos of the IabHLH domain. (**C**) The frequency of the most conserved amino acids at the respective position.

To investigate the sequence characteristics of the IabHLHs, the multiple sequence alignments of 157 IabHLH protein sequences were carried out. The results showed that the IabHLH proteins contained the typical conserved structure of basic-helix-loop-helix (Figure 4A,B). A total of 16 amino acids had values of frequency greater than 50%. Of these, the highly conserved amino acids with a frequency greater than 85% were identified within the basic region (including Glu-5, Arg-8, and Arg-9), helix 1 region (including Leu-19 and Pro-24), and helix 2 region (Leu-51).

### 2.5. Conserved Motifs, and Exon-Intron Structures of IabHLH Genes

**Figure 5:**
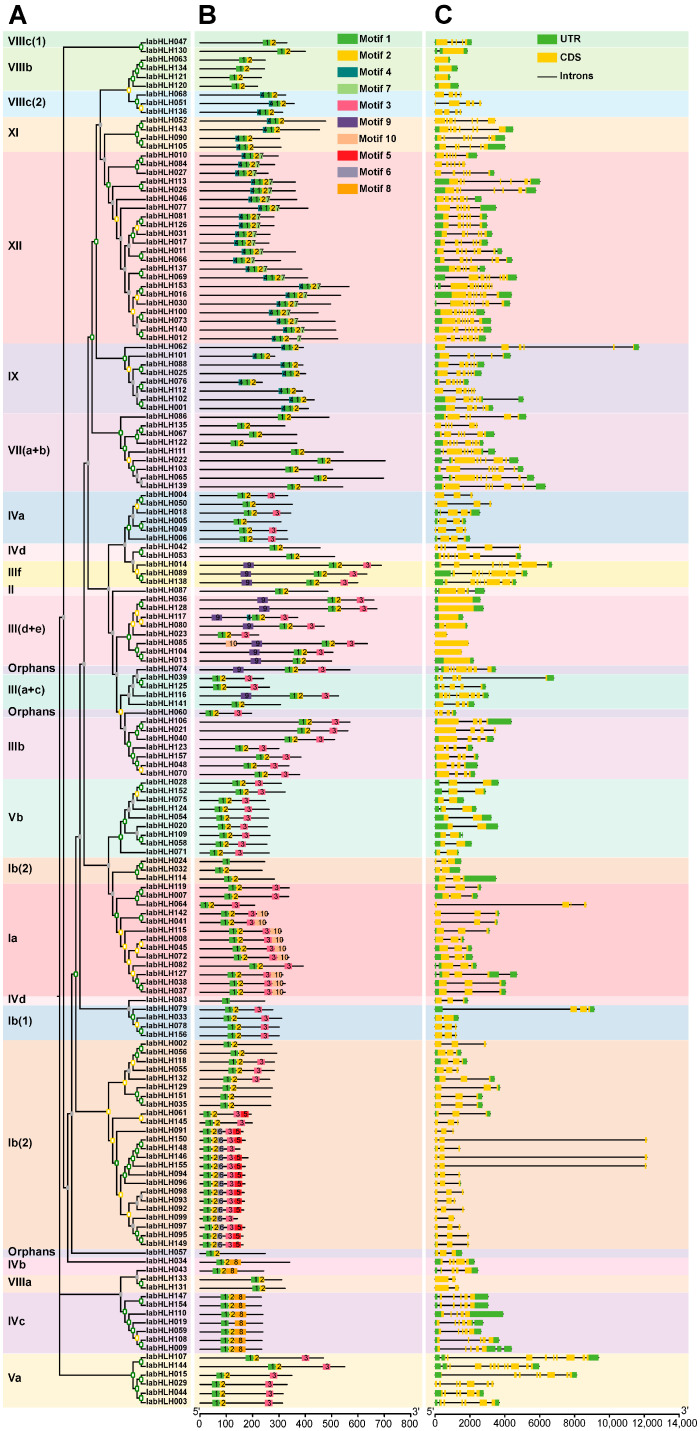
Phylogenetic tree, conserved motif, and exon-intron structure of IabHLHs. (**A**) The phylogenetic tree containing 157 IabHLH protein sequences; (**B**) distribution of conserved motifs within IabHLH proteins. The rectangles with different colors represent different motifs; (**C**) exon-intron structure of the 157 IabHLH genes. The green rectangles indicate exons, while the orange rectangles indicate untranslated regions (UTRs). The black solid lines among the rectangles indicate introns.

To further understand the functional and structural features of the IabHLH proteins, the MEME program was performed to analyze the conserved motifs within the IabHLHs. Ten conserved motifs were identified, of which motif 1 and motif 2 were present in all IabHLHs. The IabHLHs within the same group had similar motif distributions, such as the motifs of the IabHLHs in the subfamily Va, IVc, and Ib(1), indicating their similar functions. Motif 5 was only found in the IabHLHs of the subfamily Ib(2), which may impart a specific function to these IabHLHs (Figure 5A,B). The analysis of exon-intron structures within 157 *IabHLH* genes showed that the number of introns ranged from 0 to 11 (Figure 5C, Table S1 and Figure S1). The *IabHLH144* in the subfamily Va had the highest number of introns, while a total of 11 *IabHLH* genes in the subfamilies III(d+e) and VIIIb were not disrupted by an intron. Most *IabHLH* genes from the same subfamily had similar exon-intron structures.

### 2.6. Analysis of Gene Duplication Events of IabHLH Genes

**Figure 6:**
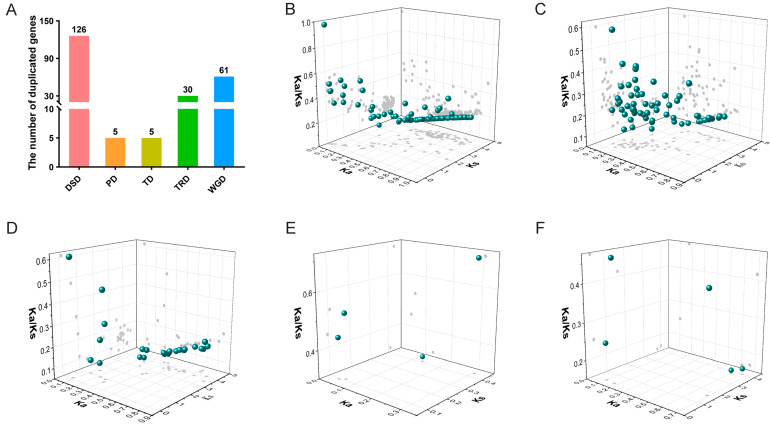
Gene duplication events of *IabHLH* genes. (**A**) The number distribution of *IabHLH* duplicated gene pairs in five duplication events. (**B**–**F**) The Ka, Ks, and Ka/Ks value distribution of the duplicated gene pairs in five gene duplication events. The DSD, WGD, TRD, PD, and TD events were from (**B**–**F**), respectively. More information concerning the duplicated genes in five gene duplication events was shown in Table S4.

To explore the potential driven force of the *IabHLH* gene family expansion, the DupGen_finder was performed to analyze gene duplication events, including WGD (whole-genome duplication), TD (tandem duplication), PD (proximal duplication), TRD (transposed duplication), and DSD (dispersed duplication). A total of 227 duplicated gene pairs derived from five duplication events were identified, with the maximum number of duplicated gene pairs derived from DSD duplication events (126 duplicated gene pairs), followed by WGD-derived duplicated gene pairs with 61. In contrast, TD and PD duplication events had the fewest number of duplicated gene pairs with only five. Moreover, we identified 30 TRD-derived duplicated gene pairs (Figure 6A and Table S4). These results suggested that the DSD and WGD duplication events played a pivotal role in the *IabHLH* gene family expansion.

To investigate the selection pressure on the *IabHLH* genes, the values of the non-synonymous substitution rate (Ka), the synonymous substitution rate (Ks), and Ka/Ks of the duplicated gene pairs derived from five replication events were calculated. As shown in Figure 6B–F, the values of Ka/Ks of all duplicated gene pairs were lower than 1, indicating these genes were subjected to the purifying selection.

### 2.7. Expression Levels of IabHLHs in Two Varieties

**Figure 7:**
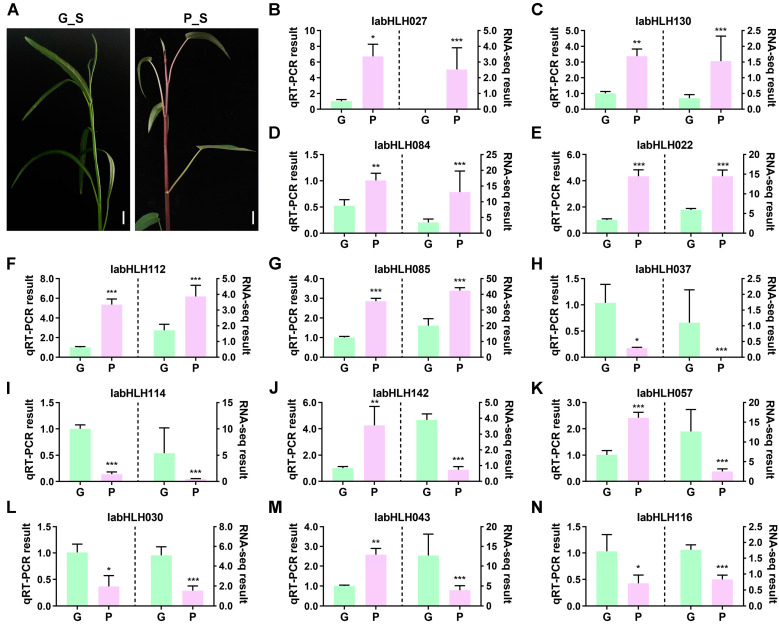
Expression analysis of the 13 DEGs. (**A**) The phenotype of two varieties. The G_S represents green-stemmed *I. aquatica*, while the P_S represents purple-stemmed *I. aquatica*. Scale bars are 2 cm. (**B**–**N**) The qRT-PCR validation of the expression trends of 13 DEGs was determined by RNA-seq. The left Y-axis and histogram indicated the relative expression levels of the *IabHLH* gene quantified by qRT-PCR. The right Y-axis and histogram indicate the FPKM values of the *IabHLH* gene obtained by RNA-seq datasets. Statistical analysis was performed using Student’s *t*-test. Data are shown as mean ± SD of three biological replicates. The *, **, and *** indicate *p* < 0.05, *p* < 0.01, and *p* < 0.001, respectively.

The purple-stemmed *I. aquatica*, which is abundant in anthocyanins, exhibits distinct phenotypic differences relative to the common green-stemmed *I. aquatica* (Figure 7A). To explore the role of the *IabHLH* genes in anthocyanin accumulation in the stems of *I. aquatica*, we analyzed the RNA-seq datasets from the purple-stemmed and green-stemmed varieties. Herein, the expression levels of 157 *IabHLH* genes in two varieties were quantified by calculating gene FPKM values according to the RNA-seq datasets. At this end, the *IabHLHs* with expression levels that met the criteria (|log_2_FC| > 1, FDR < 0.05, and *p*-value < 0.05) were considered differentially expressed genes (DEGs). A total of 13 DEGs were identified between the two varieties, including six upregulated DEGs (priority of the fold change: *IabHLH027* > *IabHLH130* > *IabHLH084* > *IabHLH022* > *IabHLH112* > *IabHLH085*) and seven downregulated DEGs (priority of the fold change: *IabHLH037* > *IabHLH114* > *IabHLH142* > *IabHLH057* > *IabHLH030* > *IabHLH043* > *IabHLH116*) in the purple-stemmed *I. aquatica* compared to the green-stemmed *I. aquatica* (Table S5). Of these, the log-transformed fold change of *IabHLH027* belonging to subfamily XII between the two varieties was the highest, with 11.3, suggesting its potential positive role in regulating anthocyanin biosynthesis in *I. aquatica*. Contrarily, the *IabHLH037* belonging to subfamily Ia was significantly downregulated in purple-stemmed *I. aquatica*. The expression trends of 13 DEGs determined by RNA-seq were validated using qRT-PCR. As shown in Figure 7B–G, the expression trends of all upregulated DEGs were highly consistent with those detected by qRT-PCR. However, three downregulated DEGs including *IabHLH142* (Figure 7J), *IabHLH057* (Figure 7K), and *IabHLH043* (Figure 7M) determined by RNA-seq had opposite expression trends of those detected by qRT-PCR.

### 2.8. Analysis of Cis-Acting Elements of DEGs

**Figure 8:**
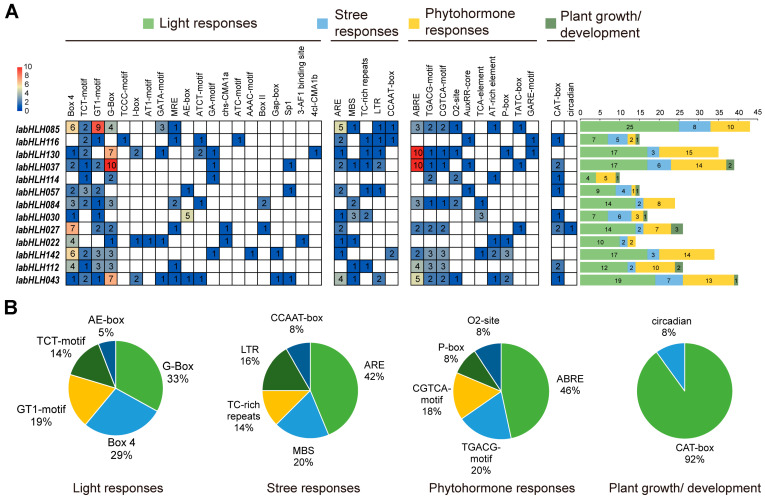
Cis-acting elements within the promoter region of the 13 DEGs. (**A**) Heatmap of the cis-acting elements. The stacked bars on the right side indicate the number distribution of different cis-acting elements in the 13 DEGs; (**B**) percentage of different cis-acting elements in the respective class.

To gain more information concerning the potential regulatory functions of 13 DEGs, the cis-acting elements within their promoter regions were analyzed by using the PlantCARE program. Except for the common cis-acting elements (TATA-box and CAAT-box) and ones of unknown function, a total of 338 cis-acting elements were detected in 13 DEGs, which were categorized as four classes, including stress-responsive elements, phytohormone-responsive elements, and plant growth and development-responsive elements (Table S6). Of these, the light-responsive elements were the largest, with 20 types including 172 elements, followed by the phytohormone-responsive elements, with 10 types including 104 elements, while the plant growth and development-responsive elements were the least, with only two types including 12 elements. The stress responsive-elements were 50, which were classified into five types. The number distribution of each cis-acting element differs in different DEGs. The light-responsive elements and the phytohormone-responsive elements were detected in all DEGs. Except for the *IabHLH114*, all DEGs had stress-responsive elements (Figure 8A), indicating their importance in plant stress response. The *IabHLH027* with the highest expression fold change between the two varieties was rich in light-responsive elements and phytohormone-responsive elements, suggesting a critical role in regulating light and phytohormone signaling pathways in *I. aquatica*.

In addition, the number of elements that belonged to the respective class varied greatly. The top five elements of the light-responsive elements were G-Box, Box 4, GT1-motif, TCT-motif, and AE-box. The AREs were the most abundant elements of stress-responsive elements, accounting for 42%. Among the phytohormone-responsive elements, the ABREs were the most numerous elements, accounting for 46%. Only one circadian-related element was detected in the plant growth and development-responsive elements (Figure 8B).

## 3. Discussion

Basic-helix-loop-helix (bHLH) proteins are widely found in eukaryotic organisms, which compose one of the largest transcription factor families in plants [38]. bHLH proteins play a vital role in regulating plant biological processes, including plant growth and development, metabolic activities, and various stress responses [39,40]. The increasing release of numerous plant genomic databases facilitates genome-wide identification of transcription factors. To date, large amounts of bHLH transcription factors have been identified in diverse plant species at the whole genome level, including model plants such as *Arabidopsis thaliana* [7] and *Nicotiana tabacum* [41], grain crops such as *Oryza sativa* [42] and *Zea mays* [6]; vegetable crops such as *Brassica rapa* [43] and *Solanum lycopersicum* [44], and fruit trees such as *Vitis vinifera* [45] and *Malus×domestica* [46]. Intriguingly, the number of *bHLH* genes varied among different plants, while it was not proportional to their genome sizes. Moreover, studies showed that the number of *bHLH* genes in higher plants is higher than that in lower plants [5,47], suggesting a pivotal role of *bHLHs* in higher plants. In this study, a total of 157 *bHLH* genes were detected in the *I. aquatica* genome based on its high-quality genome data [33]. Most IabHLH proteins were acidic and thermostable. All IabHLH proteins were hydrophilic, a principal property for transcription factors to perform their biological functions. Subcellular localization prediction showed that most IabHLH proteins were localized in the nucleus, while some were localized in the chloroplast, endomembrane system, and extracellular space, inferring that IabHLH proteins might play a distinct role in different plant organelles. These results laid a foundation for further exploring the biological function of *IabHLH* genes.

Studies have shown that the *bHLH* gene family was subdivided into a distinct number of subgroups in plants, probably due to the lack of definite classification criteria for low-conserved sequences outside the bHLH domain. Yang et al. identified 175 *bHLH* genes in *Malus × domestica* and classified them into 23 subgroups [46]. Sun et al. subdivided 159 bHLH genes of *Solanum lycopersicum* into 21 clades [44]. A total of 115 *bHLH* genes in *Vitis davidii* were gathered into 25 branches [8], while 94 *bHLH* genes in *Vitis vinifera* were clustered into 15 subclasses [45]. In the present study, phylogenetic analysis of bHLH sequences from *I. aquatica* and *A. thaliana* indicated that the 157 *IabHLH* genes could be subdivided into 23 subgroups, with three orphan genes. Compared to the *AtbHLH* gene family, although the members of the *IabHLH* gene family were lost in X, XIII, XIV, and XV subfamilies, the *IabHLH* genes in 11 subgroups (including Ia, Ib (1), Ib (2), III (a+c), IIIb, IVa, IVc, IVd, Va, Vb, IX, XII) have expanded, suggesting their key roles in the biological activities of *I. aquatica*. Moreover, three IabHLH proteins (including IabHLH014, IabHLH089, and IabHLH138) were found in the IIIf subfamily. Some reports showed that the *bHLHs* in *O. sativa* belonging to the IIIf subfamily were associated with the regulation of anthocyanin biosynthesis [48,49]. However, the expression levels of these three *IabHLH* genes have no significant difference between purple-stemmed *I. aquatica* and green-stemmed *I. aquatica*, probably other *IabHLH* genes participate in the regulation of purple color formation in the *I. aquatica* stems.

Similar to the previous reports, two adjacent highly conserved motifs and highly conserved gene structural patterns within each subclass were identified [5,50]. Studies have shown that Glu-5 and Arg-8/Arg-9 in the basic region of the bHLH structural domain play an important role in DNA binding, while Leu-19 and Leu-51 in the HLH region may be required for dimerization [1,51]. In the present study, the highly conserved amino acids were identified within the basic region (including Glu-5, Arg-8, and Arg-9), helix 1 region (Leu-19), and helix 2 region (Leu-51) (Figure 4).

Gene duplication events play an indispensable driving role in gene family expansion, which includes WGD, DSD, PD, TRD, and TD [52]. The expansions of the F-box gene family in *Gossypium hirsutum* were mainly impacted by the WGD and TD [53], while the WGD, TRD, and DSD play a dominant role in the expansions of the *P-type ATPase* gene family in *Pyrus bretschneideri* [54]. Similar to previous studies on *BAHD* and *MYB* gene families [52,55], the DSD and WGD made significant contributions to the expansions of the *IabHLH* gene family. The selection pressure could be estimated by calculating the Ka/Ks value. The Ka/Ks value of one, greater than one, and less than one indicated neutral evolution, positive selection (Darwinian selection), and negative selection (purifying selection), respectively [56,57]. The Ka/Ks values of all duplicated gene pairs in five gene duplication events were less than 1, indicating that *IabHLH* genes were subjected to the purifying selection. In addition, the syntenic relationship analysis between *I. aquatica* and other plant species verified closer evolutionary distance between similar plant taxa, suggesting the reliability of the analysis approach.

As transcription factors, the bHLH proteins directly or indirectly regulate the expression level of structural genes and thereby modulating anthocyanin biosynthesis. In grape berries, the expression level of *VdbHLH037* was highly correlated with anthocyanin accumulation in different varieties. Transgenic expression of *VdbHLH037* into *Arabidopsis* resulted in significant upregulation of structural genes involved in anthocyanin biosynthesis and high accumulation of anthocyanins. In strawberry fruits, FvbHLH9 positively regulates light-dependent anthocyanin biosynthesis by forming a heterodimer with HY [58], while in rapeseed, BnbHLH92a impinges on the biosynthesis of anthocyanins by interacting with the BnTTG1 [59]. In the present study, we found that the expression level of 13 *IabHLH* genes exhibited significant differences between purple-stemmed *I. aquatica* and green-stemmed *I. aquatica*. Of these, six DEGs were dramatically upregulated in purple-stemmed *I. aquatica* compared to green-stemmed *I. aquatica*. In particular, the *IabHLH027* belonging to the subgroup XII exhibited approximately 8-fold higher in purple-stemmed *I. aquatica* than that in green-stemmed *I. aquatica* (Figure 7B). The bHLHs of subgroup XII were correlated with positive regulation of the brassinosteroid (BR) signaling pathway [60]. Previous reports showed that BR together with other phytohormone signaling led to the enhancement of anthocyanin accumulation in plants and this process was potentially regulated by ternary MYB-bHLH-WD transcriptional complexes [61,62]. RNA-seq is widely recognized as a highly reliable and accurate method for quantifying gene expression levels, and therefore, additional qRT-PCR validation on RNA-seq results may not always be necessary when using the same samples as those used in RNA-seq. However, given the limited availability of RNA-seq samples and the potential for depletion, qRT-PCR validation of the gene of interest would be valuable when analyzing additional samples grown under the same conditions [63]. In this study, we conducted qRT-PCR analysis on additional samples and observed discrepancies in the expression trends of three downregulated differentially expressed genes (DEGs) between RNA-seq and qRT-PCR. These inconsistencies may be attributed to methodological biases that can affect the accuracy of the results obtained from each method [64,65,66]. The expression profiles of genes are closely related to their cis-regulatory elements. For instance, some *bHLH* genes in *Hordeum vulgare* containing several drought induction-associated cis-elements within the promoter regions significantly responded to drought stress stimulation [9]. The *Brassica napus bHLH* genes that possess abundant phytohormone-responsive elements were positively regulated upon exposure to more than one phytohormone, such as auxin (IAA), gibberellin (GA3), cytokinin (6-BA), abscisic acid (ABA), and ethylene (ACC) [5]. In the present study, we discovered that all DEGs had plentiful light-responsive elements and phytohormone-responsive elements, suggesting their essential function in *I. aquatica*’s response to light and phytohormone signaling. Taken together, these findings provide us valuable clues for further exploring the molecular function of *IabHLH* in regulating purple color formation in *I. aquatica* stems.

## 4. Materials and Methods

### 4.1. Identification of bHLH Genes in the I. aquatica Genome

The whole genome database of *I. aquatica* (BioProject: PRJCA002216) was used as the reference genome. The full-length sequences of AtbHLH proteins were retrieved from the TAIR (https://www.arabidopsis.org/. accessed on 1 August 2022). The BLASTP was performed to search against the *I. aquatica* proteome using AtbHLHs as queries. The HMM (Hidden Markov Model) profile of the bHLH domain (PF00010) was downloaded from the Pfam database (http://pfam-legacy.xfam.org/family/PF00010. accessed on 1 August 2022), which was used to retrieve putative *bHLH* genes from the *I. aquatica* genome. The results of the two searches were integrated to obtain candidate *IabHLH* genes. The online program CDD (https://www.ncbi.nlm.nih.gov/Structure/cdd/wrpsb.cgi. accessed on 8 August 2022), SMART (http://smart.embl-heidelberg.de/. accessed on 7 August 2022), and Pfam (http://pfam-legacy.xfam.org/search#tabview=tab1. accessed on 7 August 2022) were used to verify the structure of candidate *IabHLH* genes, while the redundant sequences and the sequences without bHLH structural domains were manually deleted.

### 4.2. Analysis of Physicochemical Parameters of IabHLH Proteins

The physicochemical characteristics of the IabHLH proteins were analyzed using the ExPASy program (http://web.expasy.org/protparam/. accessed on 22 November 2022), including the number of amino acids, molecular weight (kDa), theoretical isoelectric point (pI), instability index (Ii), aliphatic index (Ai), and grand average of hydropathicity index (GRAVY). Subcellular localization prediction of IabHLHs was carried out using BUSCA (http://busca.biocomp.unibo.it/. accessed on 13 January 2023).

### 4.3. Analysis of Phylogenetic Relationships of IabHLHs and AtbHLHs

The AtbHLH protein sequences without conserved bHLH domain were excluded [43,60,67,68]. The ClustalW2.0 program with default parameters was used to perform multiple sequence alignment of IabHLHs and AtbHLHs, and then the phylogenetic tree was constructed using the neighbor-joining (NJ) method within MEGA-X (bootstrap value: 1000) [69]. The phylogenetic tree was visualized using the online tool Evolview-v2 [70] (https://evolgenius.info/. accessed on 13 January 2023). The TBtools (version 1.108) software was used to visualize the sequence logo of the IabHLH domain.

The SWISS-MODEL (https://www.swissmodel.expasy.org/. accessed on 14 January 2023) homology modeling was performed to predict protein tertiary structures. The PyMOL (version 4.6) was used to visualize the protein tertiary structural models [71].

### 4.4. Analysis of Conserved Motif, Gene Structure, and Cis-Acting Elements

The conserved motif within IabHLH proteins was analyzed using the online program MEME (https://meme-suite.org/meme/tools/meme. accessed on 10 December 2022). The GFF annotation file of *I. aquatica* was used to obtain the intron-exon distributions of the *IabHLHs*.

The promoter sequences of the *IabHLH* genes (the upstream 2000 bp sequences from CDS of *IabHLH* genes) were extracted using TBtools based on the full-length DNA sequences of the *I. aquatica* genomes. The PlantCare online database (http://bioinformatics.psb.ugent.be/webtools/PlantCare/html/, accessed on 1 January 2023) was used to predict the cis-acting elements within promoter regions of *IabHLHs*. The results were visualized using TBtools.

### 4.5. Analysis of Chromosomal Distribution, Collinearity, and Gene Duplication Events

The chromosomal distribution of *IabHLHs* and the gene density within the chromosome of *I. aquatica* were visualized using TBtools. The collinearity relationships of *I. aquatica*, *A. thaliana*, *Ipomoea batatas*, and *Oryza sativa* were analyzed using Multiple Collinearity Scan Toolkit (MCScanX), The results were visualized using the “Advanced Circos” function within TBtools [72].

Gene duplication events of *IabHLHs* were analyzed using the dupGen_finder pipeline, which includes WGD (whole-genome duplication), TD (tandem duplication), PD (proximal duplication), TRD (transposed duplication), and DSD (dispersed duplication). The MYN method within KaKs_Calculator 2.0 (https://GitHub.com/qiao-xin/scripts_for_gb. accessed on 23 December 2022) was used to calculate the value of Ka, Ks, and Ka/Ks.

### 4.6. Plant Materials and Sampling

The purple-stemmed *I. aquatica* and green-stemmed *I. aquatica* were cultivated by cutting in Hoagland nutrient solution at 28 °C with a light cycle of 16 h (light)/8 h (dark). The uniformly colored stems of two varieties were harvested after four weeks of culture and immediately frozen in liquid nitrogen for a moment, which was then stored at −80 °C until use.

### 4.7. Transcriptome Analysis of IabHLH Genes

The transcript abundance of IabHLH genes was quantified by calculating the value of FPKM (fragments per kilobase per million mapped reads) obtained from the RNA-seq data (BioProject: PRJNA814206). The genes with expression levels that met the criteria (|log_2_FC| > 1, FDR < 0.05, and *p*-value < 0.05) were considered differentially expressed genes (DEGs).

### 4.8. qRT-PCR Analysis

Total RNA was extracted from the stems of two varieties using the RNAprep Pure extraction kit (Tiangen, Beijing, China) according to the manufacturer’s instructions. The RNA quality was assessed using the microspectrophotometer K5600C (KAIAO, Beijing, China) and agarose gel electrophoresis. The cDNA was synthesized using the FastKing cDNA kit (Tiangen, Beijing, China). The gene-specific primers were designed using Primer Premier 6.0 (Table S7). The reaction mixture of qRT-PCR includes 10 μL 2xSYBR green mix (ChamQ Universal SYBR qPCR Master Mix), 1 μL cDNA, 1 μL primers (0.5 μL forward primer and 0.5 μL reverse primer), and 8 μL PCR-grade water. The qRT-PCR was conducted on the QuantStudio Real-Time Fluorescence PCR System (ThermoFisher, MA, USA). The conditions of two-step qRT-PCR are as follows: 95 °C 3 min followed by 40 cycles of 95 °C 10 s and 55 °C 30 s. The *GAPDH* gene of *I. aquatica* was used as the internal reference gene. The relative expression of the target gene was calculated using the 2^−ΔΔCt^ method. Data were plotted using GraphPad Prism 8.

## 5. Conclusions

In this study, a total of 157 *bHLH* genes were identified in *I. aquatica* at the whole genome level, which were subdivided into 23 subgroups. The *bHLHs* within the identical subgroup had similar motifs and gene structures. The DSD and WGD duplication events made significant contributions to the expansion of the *IabHLH* gene family. All duplicated gene pairs were subjected to the purifying selection. A total of 13 DEGs were identified between the purple-stemmed and green-stemmed varieties. The expression profiling revealed that the *IabHLH027* belonging to subgroup XII was dramatically upregulated in the purple-stemmed *I. aquatica* relative to the green-stemmed *I. aquatica,* suggesting a potential positive role in regulating anthocyanin accumulation in the *I. aquatica* stems. Our study provides important gene candidates for further exploring the regulatory network of anthocyanin biosynthesis of *I. aquatica*.

## Data Availability

The datasets presented in this study can be found in online repositories. The names of the repository/repositories and accession number(s) can be found in the article/Supplementary Materials.

## Supplementary Materials

The following supporting information can be downloaded at: https://www.mdpi.com/article/10.3390/ijms24065652/s1.
