# Supplementary material for: Comprehensive Analysis of bHLH Transcription Factors in Ipomoea aquatica and Its Response to Anthocyanin Biosynthesis
Source: Int J Mol Sci. 2023 Mar 15;24(6):5652. doi: 10.3390/ijms24065652 (PMC10057536; doi:10.3390/ijms24065652)
Supplement: Supplementary file 1 [file ijms-24-05652-s001.zip › Supplementary Figure_revised.pdf]

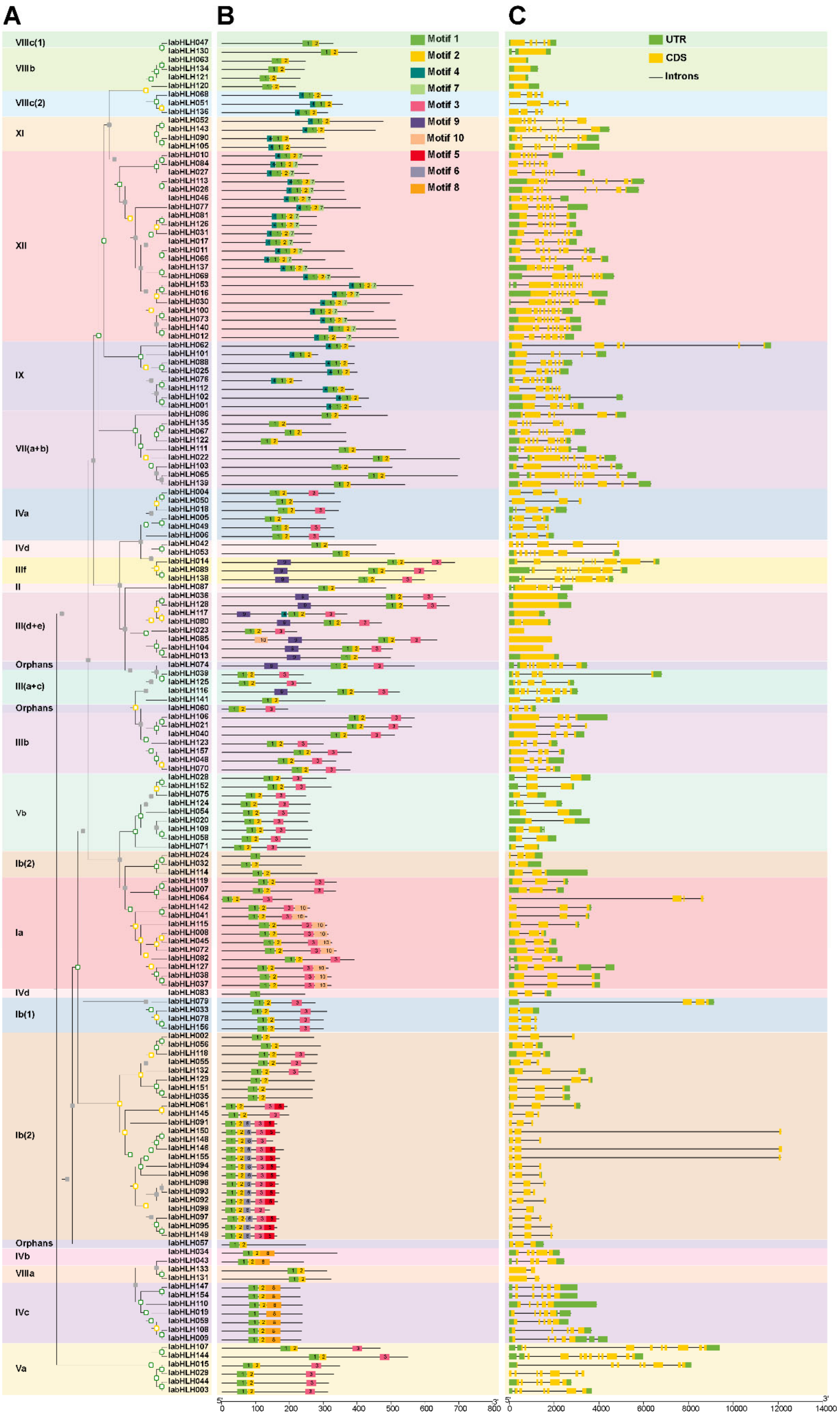

A
B
C
Motif 1
Motif 2
Motif 4
Motif 7
Motif 3
Motif 9
Motif 10
Motif 5
Motif 6
Motif 8
UTR
CDS
Introns
VIIIc(1)
VIIIb
VIIIc(2)
XI
XII
IX
VII(a+b)
IVa
IVd
IIIf
II
III(d+e)
Orphans
III(a+c)
Orphans
IIIb
Vb
Ib(2)
Ia
IVd
Ib(1)
Ib(2)
Orphans
IVb
VIIIa
IVc
Va
0
100
200
300
400
500
600
700
800
0
2000
4000
6000
8000
10000
12000
14000

Figure S1. Phylogenetic tree, conserved motif, and exon-intron structure of IabHLHs. (A) The phylogenetic tree containing 157 IabHLH protein sequences; (B) Distribution of conserved motifs within IabHLH proteins. The rectangles with different colors represent different motifs; (C) Exon-intron structure of the 157 *IabHLH* genes. the green rectangles indicate exons, while the orange rectangles indicate untranslated regions (UTRs). The black solid lines among the rectangles indicate introns.
